# A Strategic and Worldwide Cooperative Challenge Required for the Next Generation of Platelet Concentrates

**DOI:** 10.3390/ijms23073437

**Published:** 2022-03-22

**Authors:** Tomoyuki Kawase

**Affiliations:** Division of Oral Bioengineering, Niigata University Graduate School of Medical and Dental Sciences, Niigata 951-8514, Japan; kawase.dent.niigata@gmail.com

Blood-derived biomaterials, which are represented by platelet-rich plasma (PRP) or more recently by platelet-rich fibrin (PRF), have been used in regenerative therapy for almost 30 years [1]. Recently, these biomaterials are not limited to tissue regeneration but are have also been positively considered for use as anti-inflammatory agents, anti-bacterial agents, or analgesics among many clinicians. However, excessive expectations observed in the early stage have almost disappeared, and instead skeptical opinions have often been expressed especially among heavy users. As is evident in the results of recent randomized controlled trials (RCTs) and meta-analyses, it has appeared that clinical outcomes vary with individual cases. It implies that clinical outcomes are not always obtained as expected in routine regenerative therapy using PRP.

To date, because of the higher complexity, either the bioactive molecules contained in PRP or the mechanism of PRP action have not fully been clarified. Judging from the accumulated data, it is evident that bioactive molecules provided by PRP do not primarily activate cells surrounding the injury site to induce tissue regeneration. As described in more detail previously [2], PRP initially and primarily introduces blood vessels into the injury site and also acts on endothelial stem or progenitor cells to induce vascular regeneration (Figure 1a) [1]. 

What this diagram points out is the necessity of the host’s fundamental regenerative potential. For example, if a sufficient number of mesenchymal stem cells (MSCs), which are defined as multipotent stromal cells that can differentiate into a variety of cell types, are not generated in bone marrow or do not exist in peripheral tissues for example by aging, PRP is not expected to fully exert its regenerative action. Thus, it could be interpreted that PRP’s action is influenced not only by the local but by the systemic conditions of the host. PRP therapy has often been compared to adjuvant therapy (Figure 1b). This concept is explained by the essential mechanism mentioned below: if a sufficient number of stem cells are not recruited to the injury site, medication or surgical operation should be adopted to compensate for lower regenerative potential. Exogenous addition of stem cells is another option for compensation.

However, unsuccessful PRP therapy is not limited only to the case of the host’s poor regenerative potential (Figure 2). Superficially similar outcomes could be observed when the host has higher regenerative potential: if the host’s regenerative potential is higher as observed in the younger patient, the injured tissue can be cured in reasonable intervals without PRP. Conversely, the expected outcome of PRP therapy may be hidden under such fast spontaneous tissue regeneration.

PRP’s regenerative effectiveness has been under controversy for a long time. The cause of such varied clinical outcomes has simply, but ambiguously, been attributed to “individual differences”, which contain differences of both host’s local and systemic conditions and PRP quality. In this case, what we should do next is to eliminate or minimize the individual differences to realize more predictable PRP therapy. The major projects that many PRP investigators and clinicians are expecting or currently tackling are summarized to (1) clarification of the detailed mechanisms of PRP action and biomolecules involved, (2) enhancement of PRP potency and effectiveness, and (3) prolongation of PRP action (for more details, see Figure 3).

Project #1 has been long worked on from the early stage (i.e., the middle of the 1990s); however, it has not yet reached the goal because PRP is a complex mixture of many biomolecule types. In addition, the host’s responses to PRP are thought to vary with individuals. Thus, this project should be kept working but will not be completed in a short period. Projects #2 and #3 might be considered as a set and do not necessarily indicate increases in the amounts of bioactive components or elimination of inhibitory factors. In regenerative therapy, it is theoretically possible to enhance PRP’s potency, for example by adding a component or components lacking in PRP preparations, such as MSCs. On the other hand, long-term retention of active biomolecules can be realized by prevention of rapid diffusion and degradation and control of their release using biomaterials.

All the articles published in this Special Issue meet these medical needs. Aizawa et al. demonstrated the differences in platelet distribution among PRF gels prepared by different protocols [3]. The purpose of this study was to link the effectiveness and platelet distribution in PRF matrices and optimize the preparation protocol. Thus, this purpose may be included in Project #2. Devereaux et al. demonstrated that the existence of leukocytes increases the anabolic and ergogenic effects of PRP on human fibroblasts [4]. This study meets the purpose of Project #2. Nakama et al. demonstrated in the animal study that oral administration of losartan, an antagonist of angiotensin II receptor, does not reduce TGF-β1 in leukocyte-poor PRP but increases the content of CD31^+^ cells, i.e., endothelial cells, in bone marrow cell populations [5]. This study provides the data indirectly contributing to Project #2. Al-Maawi et al. demonstrated in the in vitro study that Osteopore^TM^, a bioresorbable polycaprolactone mesh, enhances PRF’s potential for bone regeneration [6]. This study meets the purpose of Project #3. Olmos Calvo et al. demonstrated hyperacute serum, a cell and fibrin-free solution that is obtained after pressing out fibrin clots, retains its regenerative potential after the freeze-drying process [7]. This may be related to Project #3. Egle et al. reviewed recent studies regarding biomaterials fabricated for better-controlled release of growth factors in PRF [8]; this article meets the purpose of Project #3.

Besides these research topics, as shown in the study of Aizawa et al. [3], optimization and standardization of the preparation protocol are very important for maintaining the quality at constant levels. PRP is a medicinal product and thus its quality should be strictly controlled in the manufacturing process and ensured before shipping. However, because many clinicians lack such a sense of product manufacturing, the quality of individual PRP preparations is not ensured in clinical settings. Under such problematic conditions, it is not difficult to imagine how much individual clinical outcomes differ from one another. Furthermore, it is nonsense to collect clinical data obtained by apparently different PRP preparations for meta-analyses.

Looking back in the past, PRP research has been left behind its clinical use, and thus, to date, the biomedical fundamentals supporting its clinical use, i.e., evidence, have not strongly been established [9]. Therefore, I would emphasize that PRP research should be concentrated on basic, pre-clinical aspects much more than large-scale, multi-institutional RCTs. I would be happy if this Special Issue functions as an active platform sharing the latest data and opinions.

## Figures and Tables

**Figure 1:**
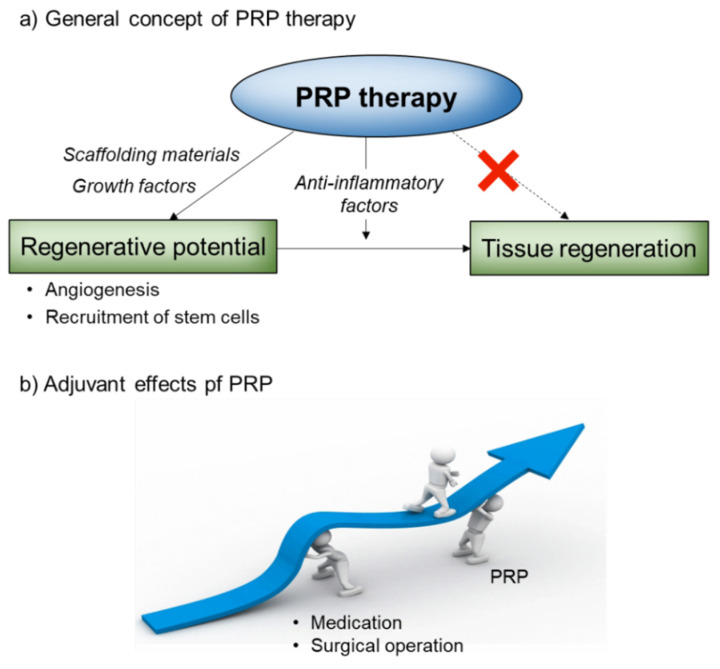
A scheme of mechanisms of PRP action. (**a**) A general concept for the scenario of PRP-induced tissue regeneration. (**b**) A proposed adjuvant effects of PRP especially in the case of lower spontaneous regenerative potential.

**Figure 2:**
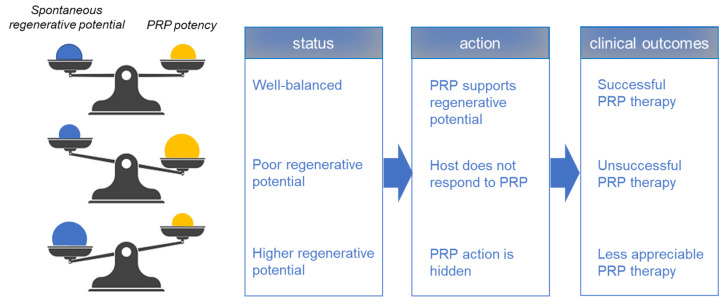
A theoretical scheme for the balance between host’s spontaneous regenerative potential and PRP potency and the effects of its balance on the success of PRP therapy.

**Figure 3:**
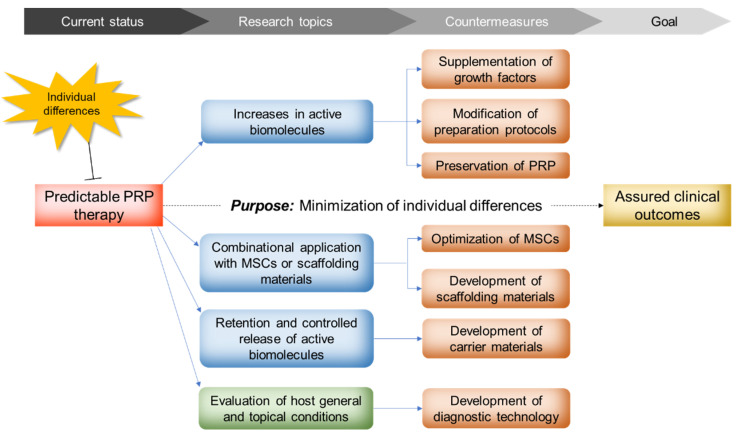
Current major research topics and their countermeasures to overcome PRP’s shortcomings.
